# Role of abbreviated non-contrast-enhanced MR-enterography in the evaluation of Crohn's disease activity and complications as an alternative for full protocol contrast-enhanced study: A systematic review and meta-analysis

**DOI:** 10.1016/j.redii.2023.100030

**Published:** 2023-04-28

**Authors:** Payam Jannatdoust, Parya Valizadeh, Mahshad Razaghi, Maedeh Rouzbahani, Amirbahador Abbasi, Arvin Arian

**Affiliations:** a School of Medicine, Tehran University of Medical Science, Tehran, Iran; b Student Research Committee, Shiraz University of Medical Sciences, Shiraz, Iran; c Advanced Diagnostic and Interventional Radiologic Research Center (ADIR), Imam Khomeini Hospital, Tehran University of Medical Sciences, Tehran, Iran

**Keywords:** Magnetic resonance imaging, Magnetic resonance enterography, Abbreviated protocol, Crohn's disease, DWI

## Abstract

**Background:**

Crohn's disease (CD) is a chronic disorder that often starts at a young age and involves periods of remission and relapse. Prompt diagnosis of relapses through screening is crucial due to the potential morbid complications of untreated active inflammation. Magnetic resonance enterography (MRE) is a noninvasive technique to screen for active inflammation. The standard protocol involves intravenous injection of contrast agents with potential side effects. Some abbreviated non-contrast-enhanced MRE protocols are proposed as alternatives for conventional MRE to identify active inflammation. Currently, there is controversy regarding the applicability and accuracy of these protocols. This study aims to describe and compare these protocols and evaluate their accuracy in detecting active inflammation and CD complications.

**Methods:**

Results from a systematic search of three databases in August 2022 were queried and screened by abstract and full text. Eligible studies were qualitatively and quantitatively analyzed by diagnostic test accuracy meta-analysis.

**Results:**

59 studies entered the systematic review, and 37 were eligible for meta-analysis. Diffusion-weighted imaging (DWI) and fast T2-weighted (T2w) sequences were most frequently used in abbreviated protocols and showed non-inferior accuracy compared to the full protocol in detecting active inflammation. ADC and qualitative DWI had pooled sensitivity of 90% (CI: 82–95%) and 89% (CI:82–93%) and pooled specificity of 94% (CI: 88–97%) and 89% (CI: 79–94%), respectively for detecting active inflammation. Moreover, T2w and combined T2w+DWI sequences had pooled sensitivity of 80% (CI: 64–90%) and 76% (CI: 61–86%) and pooled specificity of 90% (CI: 80–95%) and 87% (CI: 74 – 94%), respectively. Unenhanced protocols show relatively poor diagnostic accuracy in detecting penetrating complications of CD. Magnetization transfer imaging (MTI) has demonstrated excellent accuracy in detecting fibrosis. High heterogeneity was observed in all subgroups, and accuracy was reported to be highly operator dependent in most studies.

**Conclusion:**

An abbreviated protocol consisting of DWI and fast T2w imaging can potentially replace the full protocol MRE. Full protocol MRE will still have its role in identifying penetrating complications. MTI should be indicated in case of suspected fibrostenotic disease.

## 1 Introduction

Crohn's disease (CD) is a chronic inflammatory disorder predominantly involving the small and/or large bowl with episodes of relapse and remission, commonly diagnosed during the second to fourth decades of life [1]. Despite effective medical treatment, subclinical inflammation may persist in the gut mucosa leading to unpredictable symptomatic relapses [2]. Developing objective markers to detect subclinical inflammation would facilitate accurate diagnosis and effective treatment [3]. Regular imaging analysis is necessary for the timely detection of active inflammatory lesions, contributing to modifying treatment plans and preventing morbid complications [4,5].

While endoscopic and histopathological exams have been the gold standard in identifying inflammatory bowel wall lesions primarily upon diagnosis, their role in detecting relapsing inflammation is now limited due to invasiveness and the inability to examine the small bowel accurately. Therefore, newer guidelines have recommended cross-sectional radiologic modalities, such as computed tomographic enterography (CTE), magnetic resonance enterography (MRE), and small bowel ultrasound (US), for studying active lesions in CD patients [5,6]. Due to ionizing radiation concerns, CTE is not preferred for frequent CD relapse screening [7]. While US modalities are safer than CTE, their diagnostic accuracy highly depends on the operator [8,9]. Consequently, MRE is now the preferred method for bowel surveillance due to its lack of radiation, high contrast resolution, and ability to simultaneously study the small and large bowels [5,10].

The distinctive indicators of active inflammation detected by MRE include signs of the mural, mucosal, and stratified hyperenhancement patterns in contrast-enhanced (CE) sequences, as well as bowel wall thickening and abnormal intensity signals in non-CE sequences. The Magnetic Resonance Index of activity (MaRIA) scoring system is a well-known criterion that quantitatively assesses these signs [11,12]. In T2-weighted (T2w) and diffusion-weighted imaging (DWI) sequences, ulceration, abscess formation, fistula and sinus tract formation, comb sign, and diminished diffusion are indicators of active inflammation [13]. However, the current MRE protocol has certain drawbacks. The complete protocol imaging relies on the IV injection of GBCA, which is not recommended for patients with renal impairment or during pregnancy. Additionally, there are concerns about the long-term harmful effects of repeated gadolinium-based contrast agent (GBCA) administration, as it can deposit in the brain and bones, especially in children who may undergo surveillance screening MRE dozens of times throughout their lives [14,15]. Furthermore, the standard protocol has a long acquisition time of up to 45 min, which increases the cost and decreases compliance, particularly in younger patients [16,17].

To overcome the drawbacks stated earlier, it is recommended to use a shortened MRE protocol with an emphasis on fast T2-w sequences. Half-acquisition turbo spin-echo (HASTE) [18] and balanced steady-state free precession or true fast imaging steady-state free precession (bSSFP/trueFISP) techniques are favored due to their ability to effectively display the bowel wall while also having a shorter acquisition time, which helps to avoid temporal artifacts [16,19]. Moreover, consecutive bSSFP can form animated videos to detect diminished bowel wall motility within inflamed segments [20,21]. Furthermore, DWI sequences can enhance diagnostic accuracy without requiring GBCA and with minimal acquisition time, making them a valuable addition to abbreviated protocols. Other innovative sequences, such as magnetization transfer imaging (MTI), are being investigated for their potential to identify fibrostenotic lesions in patients with CD [22].

While some alternative sequences have been deemed comparable to the full protocol MRE, there is ongoing controversy around the reliability of their diagnostic value. As a result, there is a lack of consensus on which sequences would be optimal for use in an abbreviated protocol. Therefore, this systematic review and meta-analysis aims to compare the diagnostic abilities of different alternative sequences comprehensively.

## 2 Methods

The study was designed as a systematic review for diagnostic test accuracy (DTA) and consisted of qualitative reporting and DTA meta-analysis for applicable sequences.

### 2.1 Search methods

To retrieve all publications regarding the use of non-contrast-enhanced MRE protocols, we applied a thorough systematic search in 3 primary electronic databases for medical publications (PubMed, Scopus, Web Of Science). The search process took place in July 2022. PubMed was our first searched database, and our systematic searching strategy was as follows:

*[((DWI) OR (true fisp) OR (b-ssfp) OR (bssfp) or (FIESTA) or (balanced FFE) OR ("Magnetic Resonance Imaging, Cine"[Mesh]) OR (MR motility) or (magnetization transfer) OR (HASTE) or (abbreviated protocol) or (alternative protocol) or (non contrast enhanced) or (noncontrast) or (without contrast) or (T2w) or (T2weighted)) AND ((Magnetic resonance enterography) or (MR enterography) or (MRE)) AND ((Crohn) or (Crohn's disease))]*

After searching PubMed, we adopted a similar search strategy in Scopus and Web Of Science with appropriate modifications due to technical differences in searching each of these databases. All results were exported to the reference manager to handle and remove duplicates. Mendeley Desktop software was utilized for reference management. Review Manager ((RevMan) [Computer program]. Version 5.4. The Cochrane Collaboration, 2020, was utilized for handling data for the systematic review.

### 2.2 Study selection

After removing duplicate results using Mendeley desktop built-in tool, two researchers who were blind to each other's results, P.V. and P.J., reviewed the abstracts and titles of all studies to assess the eligibility for inclusion. M.R. then reviewed the results of P.V. and P.J., and in case of disagreements, a consensus on eligibility was reached in a meeting. Eligible records were defined as original published studies assessing the applicability or diagnostic performance of non-contrast-enhanced MRE protocols (abbreviated protocols) in diagnosing active inflammation or complications of CD in adult or pediatric patients with known CD without depending on full-protocol CE-MRE. The exclusion criteria included unpublished works, conference handouts, papers with full text in languages other than English, failing to access the full text, papers that focused on adding DWI sequences to the traditional protocol without independent analysis of DWI itself, and papers that focused on diagnosing the presence of the disease rather than its inflammatory relapsing lesions or complications.

### 2.3 Data extraction

Two researchers, P.V. and P.J., reviewed the full text of each included article and extracted qualitative and quantitative results from each paper. The extracted characteristics included the examined protocol, the reference method to which it was compared, the sign it was reporting, the segments of the bowel examined, and the age group of the study population. These data and a summary of the findings of each paper are fit into a table for qualitative reporting. Furthermore, relevant articles, sensitivity, specificity, the true and false positive (T.P., F.P.), and the true and false negative (T.N., F.N.) results were extracted and entered into RevMan software for quantitative synthesis. Some papers reported the sensitivity and the specificity based on examining the whole bowel at once, whereas others reported the rates for diagnosing each affected segment; thus, the condition that the analysis was segmental or whole-bowel analysis was included as a covariate. In several articles, full protocol CE MRE was employed as the reference standard; however, this is not a gold standard and could bring bias into the reporting of sensitivity and specificity rates. Thus, each study's reference test was also recorded to perform a sub-group analysis.

### 2.4 Assessment of methodological quality

For reporting methodological quality in DTA systematic reviews, QUADAS-2 is a standard tool [23], which consists of 17 questions in 4 domains, including patient selection, index test risk of bias and applicability, reference standard test risk of bias and applicability, and study flow and timing. Every 17 questions can be answered by the yes, no, or unclear option. The QUADAS-2 feature in Review Manager was utilized to assess the included papers in our work, and quality assessment figures were created using the same software.

### 2.5 Statistical analysis

For performing the DTA meta-analysis, spreadsheets containing T.P., F.P., T.N., and F.N. values were exported from RevMan for quantitative synthesis using appropriate models. The bivariate random-effects meta-analysis model (BRMA) model was utilized for forming summary receiver operating characteristic (SROC) curves [24]. Heterogeneity was assessed visually and by  I2 statistics introduced by Zhou & Dendukuri [25]. Deek's funnel asymmetry test plot was utilized to assess publication bias in the DTA meta-analysis, and a p-value lower than 0.10 was considered significant. In order to conduct these analyses, MIDAS [26] and METADTA [27] user-made modules were used in STATA/MP 17 (StataCorp. 2021. *Stata Statistical Software: Release 17*. College Station, TX: StataCorp LLC).

## 3 Results

**Fig. 1:**
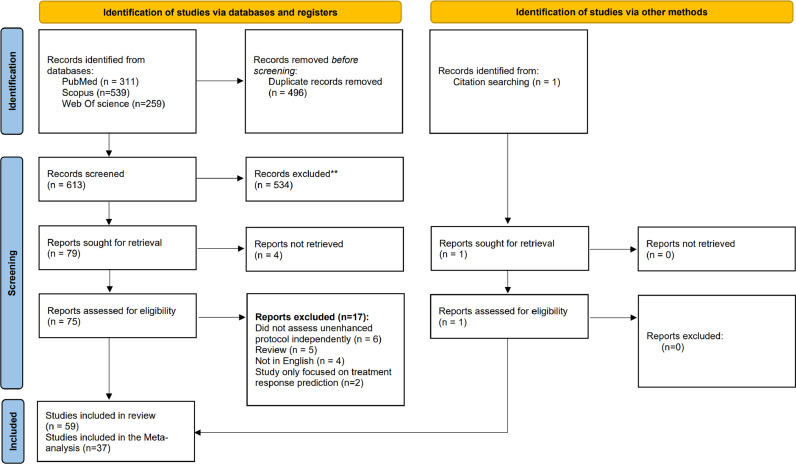
PRISMA study flow diagram.

**Fig. 2:**
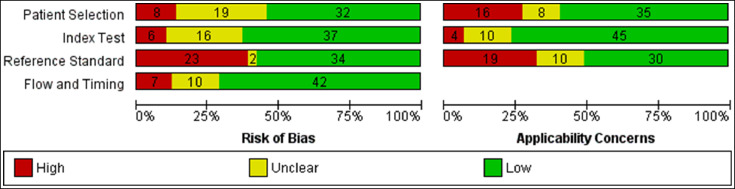
Summary of risk of bias in the 59 included studies according to QUADAS-2 criteria.

By searching the three internet databases, 613 studies were identified after removing duplicates; all of these were primarily assessed by their title and abstract. The primary title and abstract screening led to the exclusion of 534 records. Of the remaining 79 studies, the full text could not be obtained for four papers. The other 75 studies underwent full-text review, excluding 17 studies that did not match the review question, were not in English, or were review papers. Furthermore, 1 study was added by citation searching. Finally, 59 studies were included in the systematic review, and they were included in qualitative reporting. Further information about the exclusions and the reason behind them are presented in the Preferred reporting items for systematic reviews and meta-analyses (PRISMA) study flow diagram [28] (Fig. 1). The QUADAS-2 checklist assessed all the included studies for the methodological quality of the studies entering the DTA systematic review. A summary of the quality of the included papers is presented in Fig. 2. Among the 59 studies, 37 had reported enough quantitative data for the diagnostic test performance that T.P., F.P., T.N., and F.N. values could be calculated, making them eligible to enter the quantitative synthesis phase (meta-analysis). Since this study aims to measure the diagnostic ability of different MRE sequences, tests with each sequence were analyzed independently. In case a study had assessed the diagnostic abilities of more than one sequence, the data for each sequence were utilized independently in the appropriate meta-analysis. If a study had more than one radiologist examiner and reported the diagnostic results independently, mean sensitivity and specificity were calculated, and the T.P., F.P., T.N., and F.N. values were calculated accordingly.

### 3.1 Qualitative reports

The characteristics and the most noticeable results and conclusions of each included study are presented in table 1 and table 2.

**Table 1:** summary of findings of included studies that assess the diagnostic ability of unenhanced sequences in the detection of active inflammation. ADC: apparent diffusion coefficient. AUC: area under the curve. bSSFP: balanced steady-state free-precession. CD: Crohn's disease. CE: contrast-enhanced. DKI: diffusion kurtosis imaging. DWI: diffusion-weighted imaging. FCP: fecal calprotectin. FIESTA: fast imaging employing steady-state acquisition. HBI: Harvey-Bradshaw index. IBD: inflammatory bowel disease. IVIM: intravoxel incoherent motion. MARIA: magnetic resonance index of activity. MI: motility index. MRE: magnetic resonance enterography. MTI: magnetization transfer imaging. MTR: magnetization transfer ratio. Sens: Sensitivity. sMARIA: simplified magnetic resonance index of activity. SPAIR: spectral attenuated inversion recovery. Spec: Specificity. SPIR: Spectral pre-saturation with inversion recovery. SSFE: single-shot fast spin echo. STIR: Short Tau Inversion Recovery. T2w: T2 weighted. TI: terminal ileum. trueFISP: true fast imaging with steady-state precession. UC: ulcerative colitis.

Study	Alternative sequence	Reference test	Bowel sites that were examined	Assessed condition	Age group	Main results for active inflammation	Conclusion/interpretation
Fernàndez-Clotet, 2022 [57]	T2w sequences (T2-sMARIA)	ileocolonoscopy	terminal ileum and colon	therapeutic response and remission	adult	T2-sMARIA for ulcer healing: 80% Sens, 95%Spec, and high concordance with the gold standard (kappa=0.74).	T2-sMARIA shows a diagnostic accuracy similar to CE-sMARIA in all of these fields.
Jakob, 2022 [43]	DWI sequences with color coding	ileocolonoscopy	small bowel and colon	active inflammation	adult	DWI-based color-coded image: 81.3% Sens, 70.5% Spec DWI Seo score: 87.9% Sens, 71.8% Spec	DWI was as good as CE sequences in diagnosing active disease. Color-coding did not add diagnostic value
Cococcioni, 2021 [58]	CINE quantitative motility	histopathology	terminal ileum	active inflammation	pediatric	MI was lower in inflamed bowel (median:0.12) compared to normal bowel (median:0.21) (*p* = 0.02). MI has an AUC of 79.2%.	Mi decreases in active disease and can be a diagnostic marker with medium accuracy.
Djelouah, 2021 [59]	combined DWI and T2w (HASTE - trueFISP)	ileocolonoscopy	ileum and terminal ileum	postoperative recurrence of active inflammation	adult	T2+DWI: 70% Sems, 95% Spec. 0.89 AUC. All similar to CE-MRE (*p*>0.05)	DW-MRE consisting of T2 and DWI can be a reliable alternative for monitoring postoperative recurrence.
Jhaveri, 2021 [51]	T2w sequences (trueFISP)	CE-MRE - clinical score (HBI)	small bowel and colon	active inflammation, penetrating disease, fibrostenotic disease	adults	T2w bSSFP showed 97% Sens and 100% Spec for reader A and 98% Sens and 86% Spec and a perfect concordance with full protocol MRE (κ = 0.91 and κ = 0.98 for reader A and B)	bSSFP showed high diagnostic accuracy and robust concordance with MRE in assessing CD activity.
Strakšyte, 2020 [60]	DWI and ADC	ileocolonoscopy	ileum and colon	active inflammation	mixed	DWI score showed 96.9% Sens and 82.3% Spec. ADC threshold of 1.3 × $10^{-3}\;mm^{2}$/s showed 73.8% Sens and 98% Spec. MaRIA showed 97.9% Sens and 97.8 Spec.	DWI has enough accuracy in assessing the active disease. ADC shows a better specificity and a lower sensitivity.
Fang, 2020 [61]	MTI and T2w	histopathology	small bowel and colon	active inflammation, fibrosis	adult	Hyperintensity in T2w can diagnose medium to severe from mild CD activity with 87.1% Sens and 80% Spec.	T2w hyperintensity distinguishes CD activity with acceptable accuracy.
Apine, 2020 [40]	DWI and ADC	CE-MRE	terminal ileum	active inflammation	mixed	The Clermont scores assessed by DWI-STIR and SPIR correlate well with MaRIA score (rho=0.89 and rho=0.93, respectively). ADC-SPIR has a better correlation than ADC-STIR with MaRIA (rho= −0.5 and −0.001)	Clermont scores assessed by DWI-STIR and DWI-SPIR are both potential alternatives for MaRIA. ADC-STIR is not suitable for quantitative analysis.
Dreja, 2020 [62]	CINE quantitative motility	CE-MRE - Histopathology - laboratory	terminal ileum	active inflammation	adult	MI was lower in active disease (*p*<0.05) compared to healthy controls. MI has an AUC of 0.682 for differentiating active CD from the inactive phase.	MI has poor to acceptable discrimination ability for CD activity.
Kitazume, 2020 [39]	DWI and ADC	ileocolonoscopy	small bowel and colon	active inflammation	adult	AUC of DWI with *b* = 1500: 82% AUC of *b* = 800: 75.2% AUC of ADC: 81.5% AUC of CE-MRE: 83.8%	High b-value DWI has better discrimination than lower b-value DWI, similar to ADC. CE=MRE has slightly better discrimination ability ADC and DWI with *b* = 1500
Cicero, 2020 [16]	combined DWI and T2w (bSSFP - SPAIR)	CE-MRE	small bowel	active inflammation, intestinal and extra-intestinal complications	adult	There was an excellent concordance (kappa= 0.94) between the full and proposed abbreviated protocol in diagnosing active inflammation.	The proposed abbreviated protocol can be an alternative to the traditional protocol.
Cansu, 2020 [63]	combined DWI and T2w (HASTE - trueFISP)	ileocolonoscopy	terminal ileum, colon, and rectum	active inflammation	adult	There is a strong correlation between T2w&DWI-derived and CE-derived activity index (*r* = 0.97). Full protocol MRE showed 88.7% Sens and 97.9% Spec.	The scores from the proposed protocol (T2w + DWI) had a high correlation with the MaRIA score from CE-MRE.
Puylaert, 2019 [64]	DWI	ileocolonoscopy	terminal ileum	active inflammation, fistulas	n/s	DWI scores an accuracy of up to 83%, and CE-MRE has an accuracy up to 85%. DWI had significantly less confidence scores than CE-MRE(*p*<0.02).	DWI can have an accuracy almost similar to CE-MRE in case of a high-experience examiner.
Faletti, 2019 [65]	DWI and ADC	ileocolonoscopy	terminal ileum	active inflammation	adult	ADC showed an excellent discrimination power for disease activity (AUC-0.95). 1.5 × $10^{-3}\;mm^{2}$/s and 2.4 × $10^{-3}\;mm^{2}$/s were the best cutoffs for severe and moderate disease, respectively.	ADC is a valuable method for assessing CD activity and categorizing it based on severity.
Kim, 2019 [52]	combined DWI and T2w (SSFE, FIESTA)	histopathology	terminal ileum, colon, and rectum	active inflammation, penetrating disease	pediatric	The abbreviated protocol had a moderate concordance with full protocol CE-MRE (k-0,713). Their Sens and Spec were not significantly different (*p*>0.05).	An abbreviated MRE protocol consisting of pre-contrast sequences has a diagnostic ability comparable to full protocol CE-MRE.
Li, 2019 [46]	DWI and ADC and T2w	histopathology	small bowel and colon	active inflammation, fibrosis	adult	ADC values showed a moderate correlation with active inflammation score (*r*=−0.499). ADC values significantly differed between mild, moderate, and severe CD activity(*p*<0.01).	Decreased ADC can be used as a sign of the presence and severity of active CD lesions.
Yang, 2019 [42]	DWI and ADC and IVIM	ileocolonoscopy	terminal ileum, colon, and rectum	active inflammation	adult	IVIM perfusion fraction (f): 90.6% AUC ADC: 92.4%. AUC	IVIM-based (f) and ADC can be used as diagnostic tool with high discrimination rates.
Masselli, 2019 [66]	DWI and ADC	histopathology	small bowel	active inflammation	pediatric	DWI: 97.6% Sens and 96.1% Spec CE-MRE: 83.1% Sens, 96.1% Spec DWI Sens was better than CE-MRE (*p*<0.05).	CE T1w MRE can be replaced by DWI.
Yu, 2019 [67]	DWI and ADC	ileocolonoscopy	terminal ileum	active inflammation	n/s	ADC and ileum to psoas signal SR both showed high reproducibility (ICC=0.952 and ICC=0.969) ADC: 85.7% Sens and 95.7% Spec SR: up to 95.2% Sens and 95.7% Spec	SR and ADC values can quantitatively diagnose the presence of active CD lesions. SR has better sensitivity.
Durayski, 2019 [44]	DWI and ADC	histopathology	terminal ileum	active inflammation	adult	ADC: 88.8% Sens, 95% Spec DWI: 88.9%, 90% Spec	ADC and DWI can accurately identify active inflammation in CD.
Soydan, 2019 [68]	combined DWI and T2w (SSFE, FIESTA), ADC	ileocolonoscopy	ileum and colon	active inflammation	adult	ADC: 88.5% Sens, 57.1% Spec, 72.9% AUC DWI + T2 score: 88.4% Sens, 53.5 Spec.	For diagnosing moderate to severe inflammation, ADC scores have moderate diagnostic power. Combining T2w-based score and DWI does not add to accuracy.
Khachab, 2018 [69]	DWI	ileocolonoscopy	ileum and colon	active inflammation (UC and CD)	pediatric	DWI: 87% Sens, 100% Spec CE-MRI: 70% Sens, 100% Spec	DWI has similar or better sensitivity and specificity compared to CE-MRE. Accuracy for UC was less than CD.
Lanier, 2018 [70]	combined DWI and T2w (SSFE, trueFISP) and non-CE T1 weighted	histopathology	small bowel and colon	active inflammation (UC and CD), Penetrating disease	pediatric	Pre contrast: 40% Sens, 100% Spec Pre/Post Contrast: 40%Sens, 100% Spec IV contrast did not affect diagnostic accuracy or agreement rates (*p*>0.5)	Unlike IBD complications, using IV contrast does not affect the diagnosis of active inflammation. UC is also included in this study.
Menys, 2018 [71]	CINE quantitative motility	histopathology	terminal ileum	active inflammation	adult	Motility: 92% Sens, 71% spec MaRIA: 75% Sens, 74% Spec	Terminal ileal motility, assessed by an automatic algorithm, has higher sensitivity and similar specificity to MaRIA.
Huang, 2018 [41]	DWI and ADC and DKI	CE-MRE	colon	active inflammation	mixed	DKI $K_{app}$: 89.4% Sens, 95% Spec ADC: 86.4% Sens, 90% Spec	$K_{app}$ Index, assessed by DKI, has better accuracy compared to ADC.
Abd-El Khalek, 2018 [72]	DWI and ADC	CE-MRE	small bowel and colon	active inflammation	adult	3T ADC: 80% Sens, 100% Spec 1.5T DWI: 93% Sens, 90% Spec No significant difference between 3T and 15 T (*p* = 0.48)	ADC and DWI have high accuracy for active disease. Using 3T does not increase accuracy.
AlSabban, 2017 [73]	DWI and ADC	Clinical scoring	small bowel, colon, and rectum	presence of disease, active inflammation (UC and CD)	pediatric	CE-MRE: 72.5% AUC DWI: 57.2% AUC ADC: 46.1% AUC	DWI and ADC do not have enough discrimination power to detect active inflammation.
Klang, 2017 [74]	DWI and ADC	video capsule endoscopy, FCP	distal small bowel	Active inflammation	adult	ADC: 89.7% Sens, 66.7%−71.4% Spec	ADC has high accuracy in identifying patients with ulcers and increased FCP.
Li, 2017 [75]	DWI and ADC	ileocolonoscopy	ileum and colon	active inflammation	mixed	ADC: 97.3% AUC DWI: 97.2% AUC T2w SI: 84% AUC CE-MRE: 93.8% AUC	ADC has the best diagnostic power in diagnosing and grading active inflammation.
Pendsé, 2017 [36]	DWI and ADC	histopathology	small bowel, colon, and rectum	active inflammation	adult	DWI: 82% Sens, 29% Spec ADC: 86% Sens, 58% Spec	DWI and AC show high sensitivity but relatively low specificity in detecting active histopathological inflammation.
Stanescu-Siegmund, 2017 [76]	DWI and ADC	CE-MRE, clinical scoring	small bowel and colon	active inflammation	n/s	ADC: 97.4% Sens, 99.2% Spec High correlation with HBI (*p* = 0.001)	ADC values have a high agreement with CE-MRE and are a good predictor of clinical status.
Rimola, 2017 [38]	combined DWI and T2w	ileocolonoscopy	colon and rectum	active inflammation	adult	T2w: 86.4% Sens, 81.2% Spec DWI: 88% Sens, 66.7% Spec Combined: 83% Sens, 90.3 Spec MaRIA: 88.1% Sens, 96.7 Spec Combined protocol has similar Sens (*p* = 0.25) and lower Spec (*p*<0.01) compared to MaRIA.	The proposed abbreviated protocol consisting of T2w and DWI sequences has similar sensitivity and lower specificity than the conventional protocol and MaRIA score.
Park, 2017 [77]	DWI	ileocolonoscopy	terminal ileum and colon	active inflammation	adult	DWI without Buscopan: 58.2% Sens, 77.7% Spec With Buscopan: 79.4% Sens, 71.2% Spec Omitting Buscopan significantly decreases sensitivity (*p*<0.001) but does not affect specificity (*p* = 0.053)	Buscopan can not be omitted from a DWI-based abbreviated protocol since it significantly affects sensitivity.
Huh, 2017 [78]	DWI and ADC	ileocolonoscopy	terminal ileum, colon, and rectum	therapeutic response and remission	n/s	DWI: 82.5% Sens, 76% Spec	DWI has high diagnostic power in detecting complete remission and can be used for treatment monitoring.
Schmid-Tannwald, 2016 [79]	combined DWI and T2w (SSFE, trueFISP/SSFP)	histopathology	small bowel and colon	active inflammation	adult	T2w: 60% Sens, 96% Spec T2w+ DWI: 67% Sens, 96 Spec T2w + CET1w: 80% Sens, 98% Spec	DWI has a lower sensitivity but similar specificity compared to full protocol MRE.
Dillman, 2016 [37]	DWI and ADC	CE-MRE	terminal ileum	therapeutic response and remission	pediatric	ADC: 58% Sens, 52% Spec (for treatment response)	ADC does not have enough accuracy for monitoring treatment response in children.
Dubron, 2016 [80]	DWI	histopathology	small bowel and colon	active inflammation (CD and UC)	pediatric	DWI: 88.1% Sens, 83.3% Spec CE-MRE: 62.5% Sens, 97.1 Spec. DWI had significantly better sensitivity (*p* = 0.004) (per patient)	DWI with free breathing has better Sensitivity than CE-MRE.
Quaia, 2016 [81]	T2w (SPAIR)	ileocolonoscopy	terminal ileum	active inflammation	adult	Unenhanced T2w: 93% accuracy Enhanced protocol + T2w: 97% accuracy	the unenhanced protocol had non-inferior accuracy compared to the combined protocol.
Seo, 2016 [82]	DWI	ileocolonoscopy	terminal ileum	active inflammation, penetrating disease	adult	DWI: 93% Sens, 67% Spec CE-MRE: 93% Sens, 67% Spec Agreement: 91.8	DWI and CE-MRE showed similar sensitivity and specificity.
Ninivaggi, 2016 [83]	DWI and ADC	histopathology	terminal ileum	active inflammation	adult	ADC: 100% Sens, 100% Spec	ADC can detect bowel segments with active inflammation with perfect accuracy.
Jesuratnam-NielSens, 2015 [35]	combined DWI and T2w (TSE, SSH) without IV and oral contrast	CE-MRE	small bowel and colon	active inflammation	adult	T2w Wall thickening: 75% Sens, 93% Spec DWI: 58% Sens, 91.5% Spec Wall thickening had a better Sens in Colon, and DWI had a better in the small bowel.	It cannot be accurate enough to fully replace CE-MRE. T2w and DWI should both be present in an abbreviated protocol.
Plumb, 2015 [84]	CINE quantitative motility	histopathology	small bowel	treatment response	mixed	Improved MI: 93.1% Sens, 76.5% Spec (For treatment response)	MI is an accurate tool for response assessment.
Kovanlikaya, 2015 [92]	DWI and ADC	histopathology	ileum and colon	fibrosis, active inflammation	pediatric	ADC was significantly lower in diseased segments compared to normal segments. (*p*<0.05)	ADC differs between active inflammation and fibrosis.
Foti, 2015 [85]	DWI and ADC	ileocolonoscopy	terminal ileum	active inflammation	adult	Qualitative DWI: 100% Sens, 100% Spec ADC was lower in active segments. (*p*<0.0001)	DWI has perfect sensitivity and specificity.
Rosenbaum, 2015 [86]	DWI and ADC	CE-MRE	small bowel	fibrosis, active inflammation	pediatric	ADC was lower in inflamed segments compared to normal ones (*p*<0.01)	ADC can differentiate inflamed from normal segments.
Guglielmo, 2015 [49]	CINE quantitative motility	histopathology	small bowel	presence of the disease, active inflammation	mixed	Peristalsis: 84% Seen, 98% Spec	CINE bSSFP has high sensitivity and specificity for diagnosing the disease and locating active segments.
Li, 2015 [19]	DWI and ADC, and T2w (HASTE)	clinical score	small bowel and colon	active inflammation	mixed	ADC: 98% AUC DWI: 90% AUC T2w wall thickness: 83% AUC T2w hyperintensity: 80% AUC CE-MRE:68% AUC	DWI and ADC have excellent diagnostic accuracy with discrimination power more than CE-MRE.
Bickelhaupt, 2014 [87]	CINE quantitative motility	CE-MRE	small bowel	active inflammation	adult	MI is lower in affected segments compared to unaffected ones (*p*<0.01)	MI is lower segments with active inflammation, but it is not clear whether or not it can be used as a diagnostic method.
Hordonneau, 2014 [88]	DWI, ADC	CE-MRE	Small bowel and colon	Active inflammation	both	DWI: 93.7% Sens, 98.7% Spec	DWI is a reliable tool for assessing active inflammation
Buisson, 2013 [89]	DWI and ADC	CE-MRE	ileum	active inflammation	n/s	DWI: 100% Sens, 92.9% Spec ADC: 82.4% Sens, 100% Spec	DWI and ADC have good diagnostic power for monitoring small bowel.
Cullmann, 2013 [20]	CINE quantitative motility	histopathology	terminal ileum	active inflammation	n/s	Grading of motility abnormality correlated with active inflammation (*r* = 0.59) and chronic inflammation (*r* = 0.051)	Grading of bowel motility according to CINE true-FISP can detect the presence of inflammation but not its acute or chronic nature.
Menys, 2012 [48]	CINE quantitative motility	histopathology	terminal ileum	active inflammation	adult	MI had a negative correlation with histopathological score (*r*=−0.52)	Decreased TI motility can be considered a biomarker of active inflammation.
Oto, 2009 [90]	DWI and ADC	histopathology	terminal ileum and colon	active inflammation	adult	DWI: 94.7% Sens, 82.4% Spec ADC: 84% Sens, 91% Spec	DWI and ADC have high accuracy in diagnosing active inflammation.

**Table 2:** summary of findings of included studies that assess the diagnostic ability of unenhanced sequences in the detection of active inflammation. ADC: apparent diffusion coefficient. AUC: area under the curve. bSSFP: balanced steady-state free-precession. CD: Crohn's disease. CE: contrast-enhanced. DWI: diffusion-weighted imaging. FIESTA: fast imaging employing steady-state acquisition. MRE: magnetic resonance enterography. MTI: magnetization transfer imaging. MTR: magnetization transfer ratio. Sens: Sensitivity. SPAIR: spectral attenuated inversion recovery. Spec: Specificity. SSFE: single-shot fast spin echo. T2w: T2 weighted. trueFISP: true fast imaging with steady-state precession.

Study	Alternative sequence	Reference test	Bowel sites that were examined	Assessed condition	Age group	Main results for active complications	Conclusion/interpretation
Jhaveri, 2021 [51]	T2w sequences (trueFISP)	CE-MRE - clinical score (HBI)	small bowel and colon	active inflammation, penetrating disease, fibrostenotic disease	adults	TrueFISP and CE-MRE concordance for penetrating disease: Kappa=0.43–49 For stenotic disease: Kappa=0.38–0.31	There was a weak agreement between TrueFISP and full protocol MRE for diagnosing penetrating or stenotic complications.
Meng, 2020 [91]	MTI	histopathology	small bowel and colon	Fibrosis	adult	Standardized MTR: 89.5% AUC Normalized MTR: 88.5% AUC MTR: 79.8% AUC	MTR has a good correlation with fibrosis scores. Standardized MTR was superior.
Cicero, 2020 [16]	combined DWI and T2w (bSSFP - SPAIR)	CE-MRE	small bowel	active inflammation, intestinal and extra-intestinal complications	adult	Perfect (100%) agreement between full and abbreviated protocols for intestinal complications. Excellent agreement for extraintestinal complications (*K* = 0.96)	DWI and T2w can diagnose intestinal and extra-intestinal complications with similar accuracy to CE-MRE.
Fang, 2020 [61]	MTI and T2w	histopathology	small bowel and colon	active inflammation, fibrosis	adult	MTR: 91.3% Sens, 92.3% Spec	MTR has good diagnostic ability in diagnosing fibrosis. Wall thickness is an indicator of both inflammation and fibrosis.
Caruso,2020 [45]	DWI and ADC	histopathology	ileum and colon	Fibrosis	adult	T2w wall thickness: 69.2% Sens, 100% Spec ADC: 72% Sens, 94% Spec	Increased wall thickness and decreased ADC can help to diagnose fibrosis. ADC has better specificity but less sensitivity.
Kim, 2019 [52]	combined DWI and T2w (SSFE, FIESTA)	histopathology	terminal ileum, colon, and rectum	active inflammation, penetrating disease	pediatric	Among 10 findings of penetrating disease in CE-MRE, only 3 were diagnosed on pre-contrast images.	Pre-contrast sequences may underestimate penetrating complications.
Puylaert, 2019 [64]	DWI	ileocolonoscopy	terminal ileum	active inflammation, fistulas	n/s	Of five fistulas diagnosed by CE-MRE, only 3 were diagnosed with DWI. DWI did not reveal any other fistulas.	DWI may underestimate fistula rates.
Barat, 2019 [56]	combined DWI and T2w (HASTE - trueFISP)	histopathology	small bowel	stenosis, fistula, abscess	adult	Senior radiologist DWI for Fistula: 80% Sens, 70% Spec Stenosis: 81% Sens, 56% Spec Abscess: 100% Sen For junior examiner, DWI sensitivity was lower than CE-MRE. (*p*<0.01)	DWI had similar accuracy for diagnosing complications compared to CE-MRE by a senior radiologists but not a junior radiologist.
Li, 2019 [46]	DWI and ADC and T2w	histopathology	small bowel and colon	active inflammation, fibrosis	adult	ADC had an AUC of 86.7% in diagnosing moderate to severe fibrosis. Difference of ADC value between fibrotic and non-fibrotic sections is not significant in the case of moderate to severe inflammation (*p* = 0.613)	ADC serves as a valuable tool for diagnosing fibrosis in CD. However, its diagnostic ability falls in case of moderate to severe inflammation.
Li, 2018 [22]	MTI, DWI, and ADC	histopathology	small bowel	Fibrosis	adult	Normalized MTR: 96.5% Sens, 91.7% Spec, 98.1% AUC ADC: 86% AUC CE-MRE: 64.6% AUC Normalized MTR did not correlate with inflammation activity (*p* = 0.74)	Normalized MTR has high discriminating power for detecting fibrosis. Decreased ADC can also be a sign of fibrosis. Unlike ADC, MTR is not correlated with inflammation.
Lanier, 2018 [70]	combined DWI and T2w (SSFE, trueFISP) and non CE T1 weighted	histopathology	small bowel and colon	active inflammation (UC and CD), Penetrating disease	pediatric	5 patents had perianal abscess or fistula in CE MRE. Only 2 of these were detectable in pre-contrast MRE.	Abbreviated contrast-less MRE protocol might underestimate CD complications including Penetrating disease.
Seo, 2016 [82]	DWI	ileocolonoscopy	terminal ileum	active inflammation, Penetrating disease	adult	Among 8 segments of penetrating complications identified by CE-MRE, DWI showed similar findings in only 5 of the segments.	DWI may be inaccurate for diagnosing penetrating complications.
Kovanlikaya, 2015 [92]	DWI and ADC	histopathology	ileum and colon	fibrosis, active inflammation	pediatric	ADC was significantly lower in fibrotic segments compared to normal and inflamed nonfibrotic segments. (*p*<0.02)	ADC differs in fibrotic segments compared to both inflamed nonfibrotic and normal segments.
Rosenbaum, 2015 [86]	DWI and ADC	CE-MRE	small bowel	fibrosis, active inflammation	pediatric	Fibrostenotic segments had lower ADC compared to inflamed segments and normal ones (*p*<0.01)	Follow up ADC can be a benevolent method in detecting fibrostenotic disease.
Ream, 2013 [93]	DWI and ADC	CE-MRE	terminal ileum	stricture, penetration	pediatric	ADC was lower in presence of stenosis (0 = 0.02) but nit penetrating disease (*p* = 0.52)	ADC and DWI can be used for diagnosing stenosis but not penetrating disease.
Pazahr, 2013 [94]	MTI	histopathology	ileum and colon	Fibrosis	adult	MTR was increased in segments with fibrosis compared to inflamed and normal segments (*p*<0.0001)	MTI is feasible and can help distinguishing fibrostenotic segments.

### 3.2 Studies concerning the detection of active inflammation using unenhanced sequences

Table 1 displays data on the diagnostic performance of unenhanced imaging protocols in the detection of active inflammation. A total of 53 studies reported the diagnostic efficacy of abbreviated protocols and unenhanced sequences in the diagnosis of active inflammation. DWI sequences and ADC measurements were the most frequently assessed sequences among the studies included in the analysis. Additionally, a number of studies evaluated the diagnostic performance of T2-weighted sequences, including bSSFP, either alone or in conjunction with DWI sequences. Moreover, eight studies investigated the use of animated CINE trueFISP images to evaluate diminished bowel motility, referred to as the frozen bowel sign, in the diagnosis of active inflammation. Although the results pertaining to the correlation between motility indices assessed by CINE trueFISP and active inflammation were intriguing, most studies concluded that this sequence lacked the discriminatory power needed for diagnosing active inflammation. Further information about each study concerning the diagnosis of active inflammation and their primary findings are available in Table 1.

### 3.3 Studies concerning the diagnostic ability of unenhanced sequences in diagnosing CD complications

The data presented in Table 2 pertains to the diagnostic ability of unenhanced sequences in detecting complications associated with Crohn's disease (CD), including fibrostenotic and penetrating complications. 8 studies were included in the qualitative analysis, which evaluated the performance of unenhanced T2w and DWI sequences in comparison to traditional CE-MRE for detecting penetrating disease. The majority of these studies reported that unenhanced MRE protocols exhibit suboptimal diagnostic accuracy for detecting various types of penetrating complications and may potentially underestimate their presence.

In addition, 11 studies were included in the analysis of the diagnostic ability of unenhanced sequences in identifying fibrostenotic disease. DWI and MTI were the most frequently evaluated protocols for this purpose. The results showed that while fibrostenotic disease is associated with restricted diffusion in DWI sequences, this feature cannot be used as a reliable tool for detecting fibrosis since similar patterns of diffusion restriction are also present in active inflammation. Furthermore, four studies analyzed the diagnostic ability of MTI in identifying fibrostenotic conditions and found that, in most cases, MTI has an excellent diagnostic performance in assessing fibrostenotic complications.

Due to the limited number of studies investigating the diagnostic efficacy of unenhanced sequences in evaluating CD complications, we were unable to conduct a meta-analysis in this area. Further information regarding the studies analyzing the assessment of CD complications is provided in table 2.

### 3.4 Quantitative synthesis of diagnostic accuracy of unenhanced sequences in the assessment of active disease

**Fig. 3:**
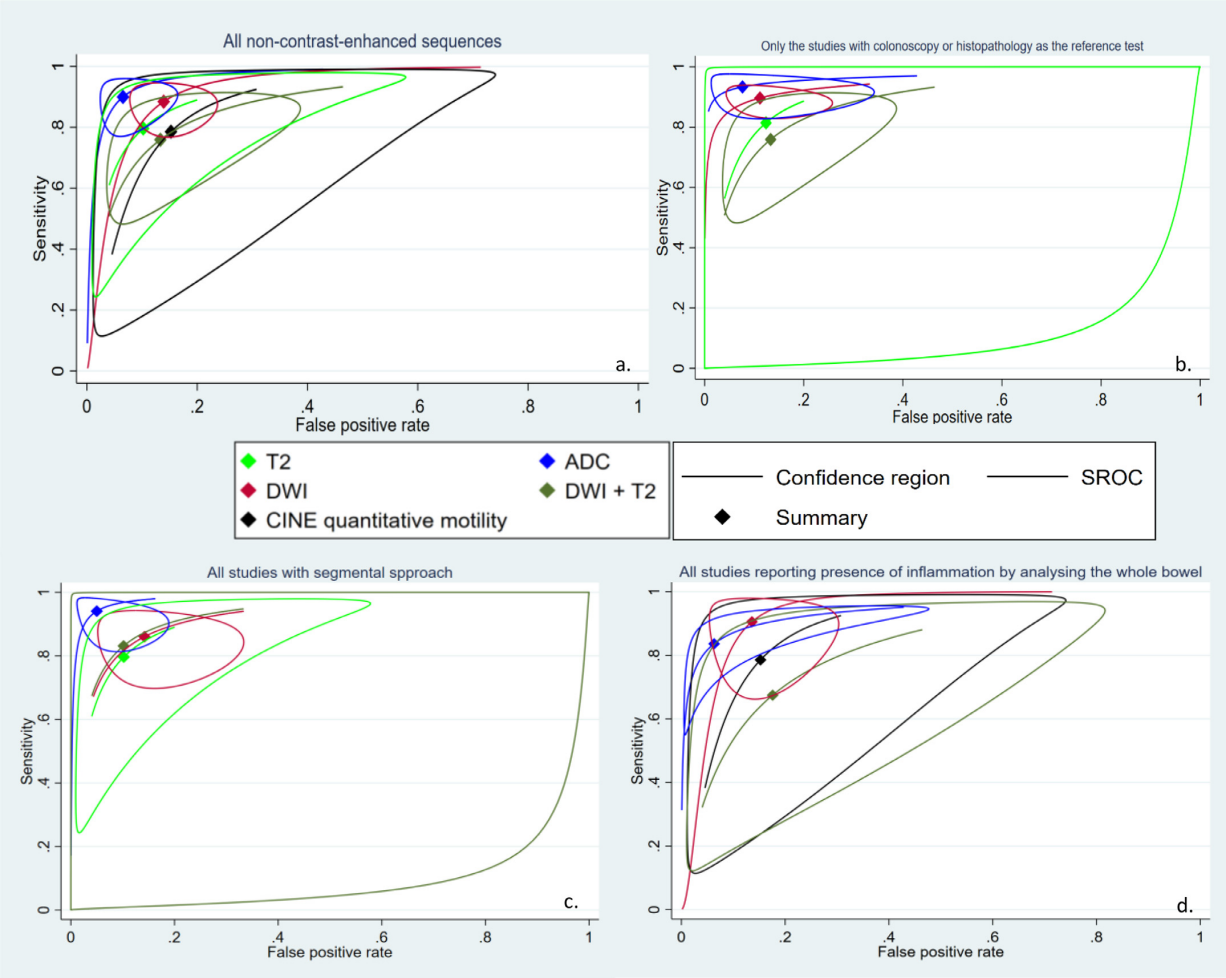
Meta-analysis of diagnostic test accuracy of all non-contrast-enhanced sequences that had been deployed in enough included studies(*n* ≥ 3) to form a summary receiver operating characteristics (SROC) curve. a. SROC curve of all non-contrast-enhanced sequences regardless of study design. b. SROC curve of all studies that used colonoscopy or histopathology as the reference standard. c. SROC curve of all studies that examined each segment of the bowel by itself by a segmental approach. d. studies that examined the presence of active inflammation in the whole bowel. ADC: apparent diffusion coefficient. DWI: diffusion-weighted imaging. SROC: summary receiver operating characteristics.

As discussed earlier, the quantitative data were analyzed in subgroups regarding the assessed protocol. Because several studies had reported the test accuracy indices of more than one MRI sequence, these studies independently entered meta-analysis for different types of imaging sequences. For instance, 20 studies were eligible to enter the meta-analysis for diagnostic accuracy of DWI sequences. Furthermore, 18 and 4 studies were eligible to enter the meta-analyses for apparent diffusion coefficient (ADC)T2 and T2-based imaging (including bSSFP/trueFISP), respectively. Moreover, several studies have reported the diagnostic abilities of an abbreviated protocol with combined DWI and T2w sequences. Thus this combined protocol was analyzed as an independent subgroup (*n* = 7). Finally, four studies reported the diagnostic abilities of quantitative bowel motility indices. In order to obtain a generalized overview of the matter, Fig. 3a depicts the SROC curve for the five categories of sequences, all stratified into one graph. Many studies had chosen tests other than colonoscopy or histopathology (i.e., full protocol MRE, clinical scoring, fecal calprotectin, …) as the reference standard to which the tests were compared, and this can be an important source of bias. Thus, Fig. 3b is also presented, which only includes studies with the gold standard tests (colonoscopy and histopathology) as their reference standard. The circumstances under which a study has computed diagnostic accuracy indices are another source of bias and heterogeneity. For instance, the estimate might be based on examining the bowel of each patient in its entirety or on individually analyzing distinct segments of the bowel. Thus, we also performed a subgroup meta-analysis based on this condition in applicable studies. Figs. 3c and 3d present the SROC curves for studies with segmental or whole-bowel analyses, respectively. Table 3 demonstrates the summary statistics on the diagnostic ability of the included unenhanced protocols.

**Table 3:** Thresholds for apparent diffusion coefficient (ADC) to distinguish active inflammation from normal bowel wall with the highest area under the curve (AUC) in the receiver operating characteristics (ROC) curve. ADC: apparent diffusion coefficient. AUC: area under the curve. ROC: the receiver operating characteristics.

Study	Reference test	ADC threshold ($10^{-3}\;mm^{2}$/s)	AUC	Sensitivity	Specificity
Yang, 2019 [42]	ileocolonoscopy	0.87	0.924	0.906	0.936
Li, 2015 [19]	clinical score	1.12	0.98	1	0.88
Strakšyte, 2020 [60]	ileocolonoscopy	1.32	0.883	0.979	0.978
Abd-El Khalek, 2018 [72]	CE-MRE	1.38	n/s	0.93	0.9
Schmid-Tannwald, 2016 [79]	histopathology	1.41	0.95	0.93	0.75
Soydan, 2019 [68]	ileocolonoscopy	1.47	0.729	0.885	0.657
Huang, 2018 [41]	CE-MRE	1.485	0.907	0.864	0.9
Stanescu-Siegmund, 2017 [76]	CE-MRE, clinical scoring	1.56	0.998	0.974	0.992
Li, 2017 [75]	ileocolonoscopy	1.59	0.974	0.972	0.843
Yu, 2019 [67]	ileocolonoscopy	1.81	0.929	0.857	0.957
Hordonneau, 2014 [88]	CE-MRE	1.9	n/s	0.937	0.96
Klang, 2017 [74]	video capsule endoscopy	1.92	0.832	0.897	0.714
Durayski, 2019 [44]	histopathology	2.1	0.9194	0.888	0.95
Ninivaggi, 2016 [83]	histopathology	2.416	1	1	1
Faletti, 2019 [65]	ileocolonoscopy	1.5 (for severe) 2.4 (for moderate)	0,95 (for severe)	0.829 (for severe) 0.8 (for moderate)	0.913 (for severe) 0.914 (for moderate)

### 3.5 DWI sequences

**Fig. 4:**
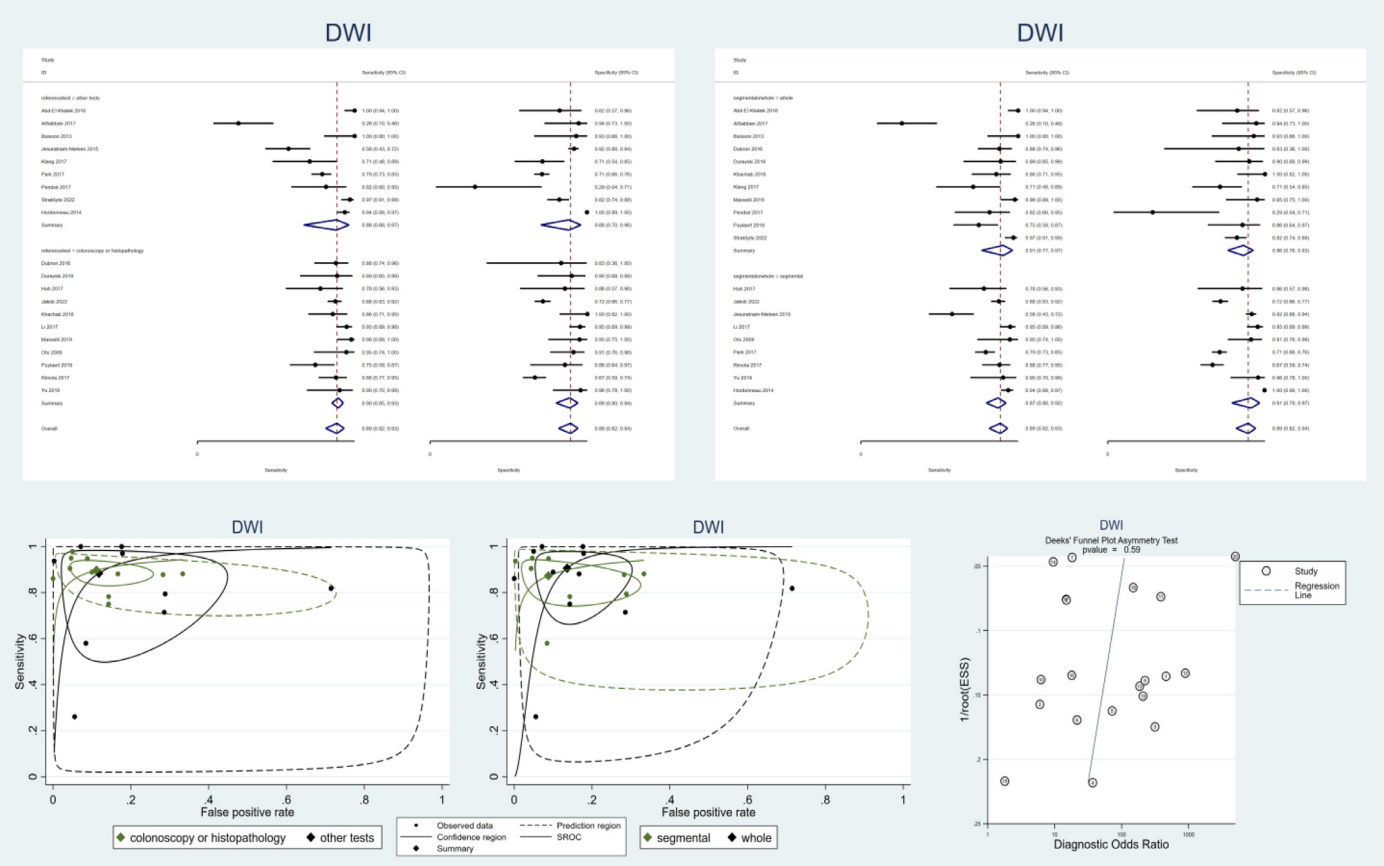
Meta-analysis of all studies of diagnostic test accuracy of all included diffusion-weighted imaging (DWI) studies. a. Forest plot of DWI studies stratified by the utilized reference test. b. Forest plot of DWI studies stratified by the study design (segmental or whole-bowel approach) c. Summary receiver operating characteristics (SROC) of DWI studies stratified by the utilized reference test. d. SROC of DWI studies stratified by the study design (segmental or whole-bowel approach). e. Deek's funnel plot asymmetry test to investigate publication bias. DWI: diffusion-weighted imaging. SROC: summary receiver operating characteristics.

Of the five analyzed modalities, DWI had the highest number of included studies (n:20). Figs. 4a and 4c demonstrate the forest plots and the SROC curves for DWI based on the reference tests. Figs. 4b and 4d demonstrate these plots based on the study design, either segmental or whole-bowel analysis. Furthermore, Fig. 4d demonstrates Deek's funnel plot for publication bias. Regarding the p-value for the asymmetry test, DWI studies have no significant publication bias.

In our meta-analysis, DWI studies on the assessment of active inflammation have a pooled sensitivity of 89% (95% CI: 82–93%), a pooled specificity of 89% (95% CI: 79–94%), and high heterogeneity ($I^{2}$ = 98%). Reference tests other than colonoscopy and histopathology may introduce bias by overestimating the test accuracy and being a significant source of heterogeneity. Subgroup analysis for studies with gold standard test as a reference test reveals a pooled sensitivity of 90% (95% CI: 85–93%), a pooled specificity of 89% (95% CI: 80–94%), and lower but still substantial heterogeneity ($I^{2}$ = 75%) proving the theory that different reference tests is a significant source of heterogeneity in our analysis. Results in segmental and whole-bowel analyses are almost similar, except that visual assessment of the SROC curve proposes that sensitivity might be underestimated when the analysis is segment based. It is important to note that even after excluding studies with low-accuracy reference tests, heterogeneity remains substantial ($I^{2}$ = 75%) as a result of factors such as the analyzed sites of the bowel, which could not be included as a covariate in our meta-analysis due to the limited number of relevant studies in each category.

### 3.6 ADC

**Fig. 5:**
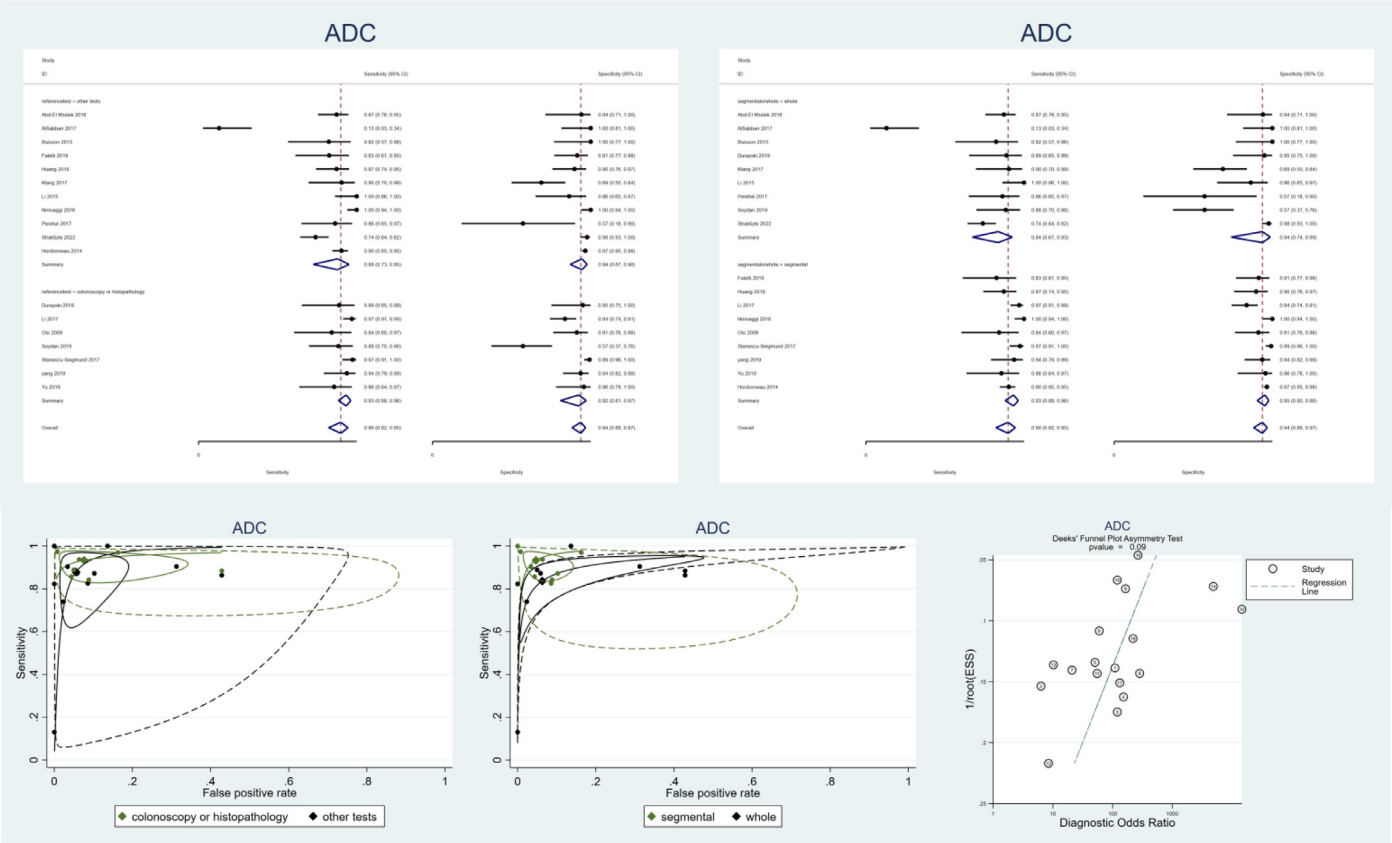
Meta-analysis of all studies of diagnostic test accuracy of all included apparent diffusion coefficient (ADC) studies. a. Forest plot of ADC studies stratified by the utilized reference test. b. Forest plot of ADC studies stratified by the study design (segmental or whole-bowel approach) c. Summary receiver operating characteristics (SROC) of ADC studies stratified by the utilized reference test. d. SROC of ADC studies stratified by the study design (segmental or whole-bowel approach). e. Deek's funnel plot asymmetry test to investigate publication bias that shows a significant publication bias (*p*<0.05). ADC: apparent diffusion coefficient. SROC: summary receiver operating characteristics.

Figs. 5a and 5c present the forest plot and the SROC plot of the 17 studies with ADC accuracy tests divided based on the reference test. Similar to DWI studies, ADC studies have also been divided based on the segment- or patient-oriented study design (5b, 5d). Furthermore, 5e demonstrates the publication bias assessment in ADC studies, which shows a potential publication bias (*p*<0.05). Thus, the results regarding the ADC accuracy might be biased and should be considered more carefully.

As demonstrated in Fig. 3a, in comparison to other modalities, ADC might have a higher diagnostic power. Quantitative analysis revealed that included ADC studies have a pooled sensitivity of 90% (95% CI: 82–95%), a pooled specificity of 94% (95% CI: 79–94%), and high heterogeneity ($I^{2}$ = 97%). Subgroup analysis for studies with gold standard test as a reference test reveals a pooled sensitivity of 93% (95% CI: 88–96%), a pooled specificity of 92% (95% CI: 81–97%), and lower but still substantial heterogeneity ($I^{2}$ = 74%).

An essential characteristic of ADC mapping is its quantitative nature. The ADC method calculates a quantitative index that can objectively predict the presence of inflammation. However, there is no consensus regarding the optimal threshold for distinguishing inflammatory from non-inflammatory sites for the ADC value. Thus, in our included studies, the different thresholds can be a source of heterogeneity. Thus, we have reported the optimal threshold values and diagnostic test characteristics, including sensitivity, specificity, and area under the curve (AUC) in the included studies (Table 4).

**Table 4:** Pooled sensitivity, specificity, and heterogeneity $\left(I^{2}\right)$ of studies with each non-contrast-enhanced sequence. "gold standard" refers to tests with ileocolonoscopy or histopathology as their reference standard. ADC: apparent diffusion coefficient. DWI: diffusion-weighted imaging. T2w: T2 weighted imaging.

	All studies		Studies with gold standard reference test			
	Sensitivity	Specificity	$I^{2}$	Sensitivity	Specificity	$I^{2}$
ADC	0.90 [0.82 – 0.95]	0.94 [0.88 - 0.97]	97%	0.93 [0.88 - 0.96]	0.92 [0.81 - 0.97]	74%
DWI	0.89 [0.82 - 0.93]	0.89 [0.79 - 0.94]	98%	0.90 [0.85 - 0.93]	0.89 [0.80 - 0.94]	75%
Combined T2w and DWI	0.76 [0.61 - 0.86]	0.87 [0.74 - 0.94]	93%	0.76 [0.61 - 0.86]	0.87 [0.74 - 0.94]	93%
T2w	0.80 [0.64 - 0.90]	0.90 [0.80 - 0.95]	91%	The number of studies was not enough		
CINE motility	0.79 [0.56 - 0.91]	0.85 [0.70 - 0.93]	91%	The number of studies was not enough		

### 3.7 T2w protocols

**Fig. 6:**
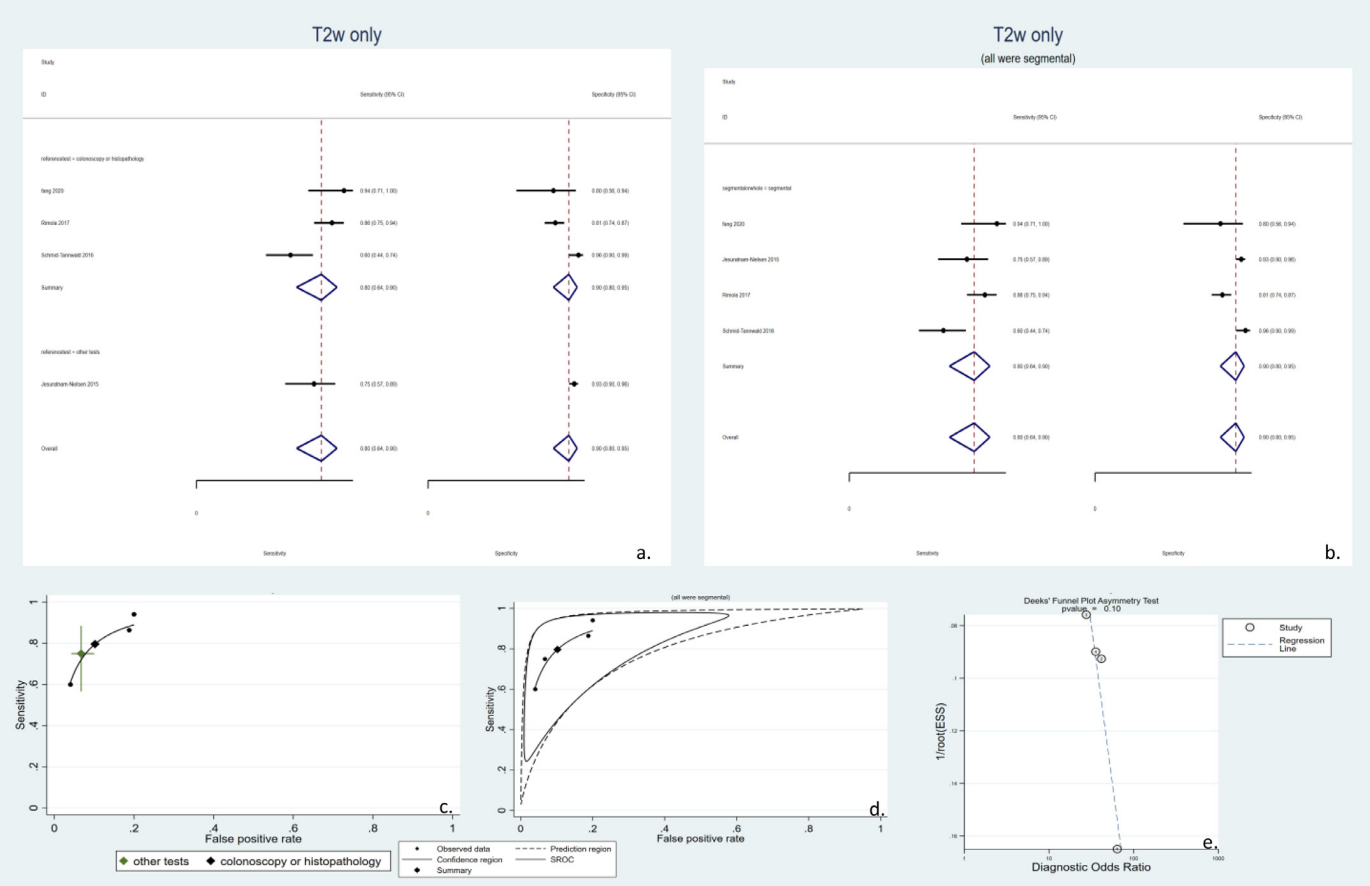
Meta-analysis of all studies of diagnostic test accuracy of included T2 weighted (T2w) studies. a. Forest plot of T2w studies stratified by the utilized reference test. Due to the presence of only one study with a reference test other than ileocolonoscopy or histopathology, meta-analysis was not possible in this category. b. Forest plot of T2w studies stratified by the study design (segmental or whole-bowel approach). All of the studies using this modality had a segmental design. c. Summary receiver operating characteristics (SROC) of T2w studies stratified by the utilized reference test. Due to the presence of only one study with a reference test other than ileocolonoscopy or histopathology, meta-analysis and drawing SROC were not possible in this category. d. SROC of T2w studies stratified by the study design (segmental or whole-bowel approach). All of the studies using this modality had a segmental design. e. Deek's funnel plot asymmetry test to investigate publication bias. SROC: summary receiver operating characteristics. T2w: T2 weighted.

Although various types of T2w sequences can be used in distinguishing active lesions, because of the small number of studies on each type of T2w sequence, this study has aimed to describe the T2w sequences as a whole. As depicted in Fig. 6, there were only four relevant studies, minimizing the statistical power to interpret the data. These studies had a pooled sensitivity of 80% (95% CI: 64–90%), a pooled specificity of 90% (95% CI: 80–96%), and high heterogeneity ($I^{2}$ = 91%). There was only 1 study with a reference test other than gold standards. Thus, we could not form an SROC graph for this subgroup (Figs. 6a, and 6c). Furthermore, all of the included studies had a segmental approach for analysis (Fig. 6b, 6d). No statistically significant publication bias was observed (Fig. 6e).

### 3.8 CINE sequences for quantitative bowel motility measurement

**Fig. 7:**
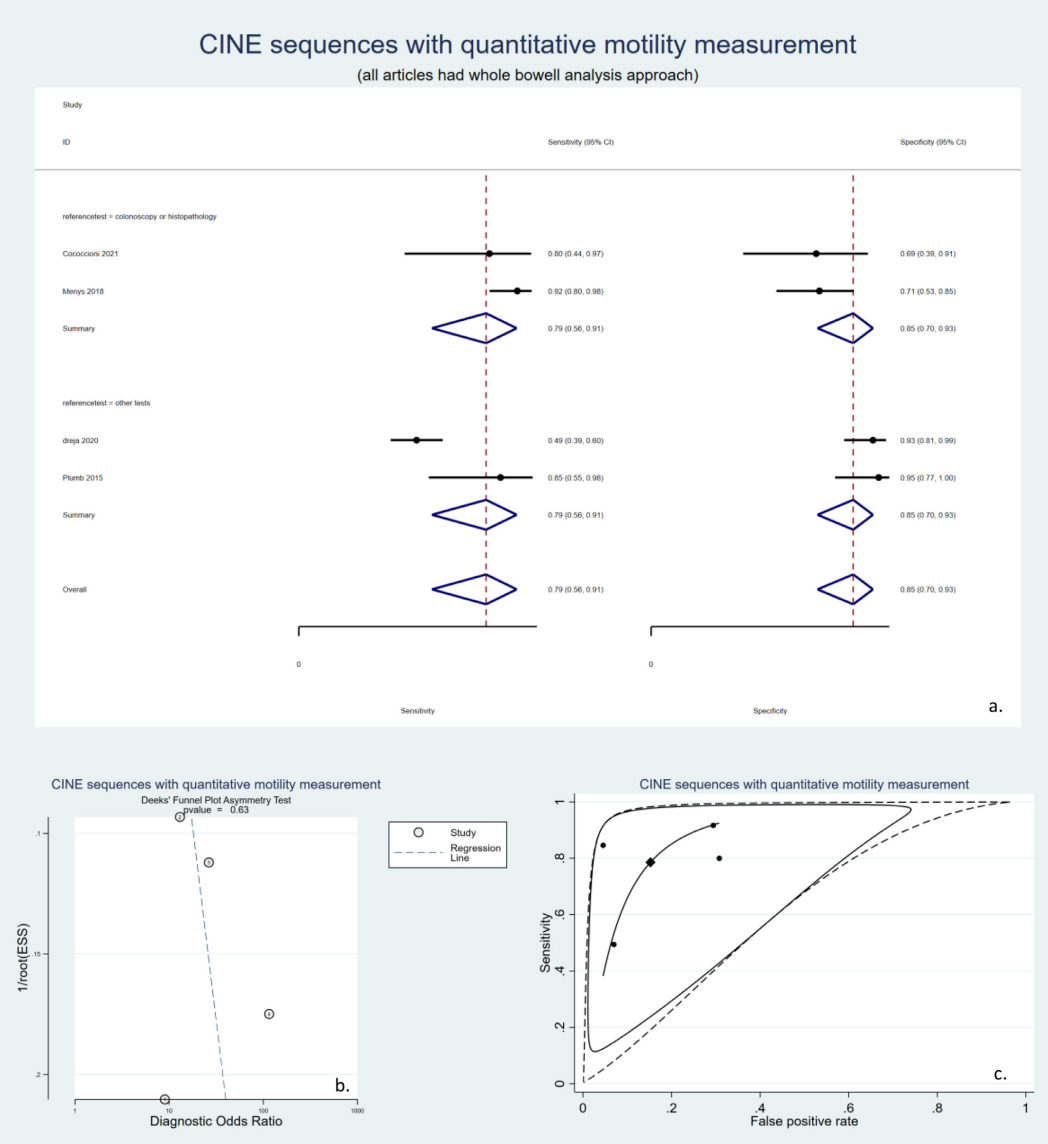
studies that used CINE sequences to quantitatively measure bowel motility. a. Forest plot of studies with quantitative bowel motility study stratified by the reference tests. All of these studies had a segmental approach. b. Deek's funnel plot asymmetry test of publication bias. c. Summary receiver operating characteristics (SROC) of included studies. Due to the small number in each category, separate SROCs could not be drawn. SROC: summary receiver operating characteristics.

CINE MRE imaging techniques are animated images based on fast T2w sequences that can be utilized to study diminished bowel mobility, named the frozen bowel sign. M.R. motility can be quantified using computer software and analyzed objectively. Although many studies have analyzed the relationship between bowel motility and the presence of inflammation, which are included in table 1, only four studies reported the sensitivity and specificity measures and were qualified to enter the meta-analysis, as depicted in Fig. 7. These studies had a pooled sensitivity of 79% (95% CI: 64–90%), a pooled specificity of 85% (95% CI: 70–93%), and high heterogeneity ($I^{2}$ = 91%). All relevant studies were based on the whole bowel. Regarding the study reference test, there were only two studies in each subgroup (Fig. 7a), preventing the statistical module from generating an SROC graph for either of the two groups. (Fig. 7b). Publication bias was not significant in these studies either (Fig. 7c).

### 3.9 Mixed protocols with combined T2w and DWI sequences

As discussed, an appropriate abbreviated protocol to replace the current full protocol might include a combination of different non-contrast-enhanced MRE sequences and a reporting system to report the results from the combination of the two sequences. Several studies have recently developed novel abbreviated protocols and have tried to evaluate the diagnostic effectiveness of the mixed protocol that combines T2w and DWI tests.

**Fig. 8:**
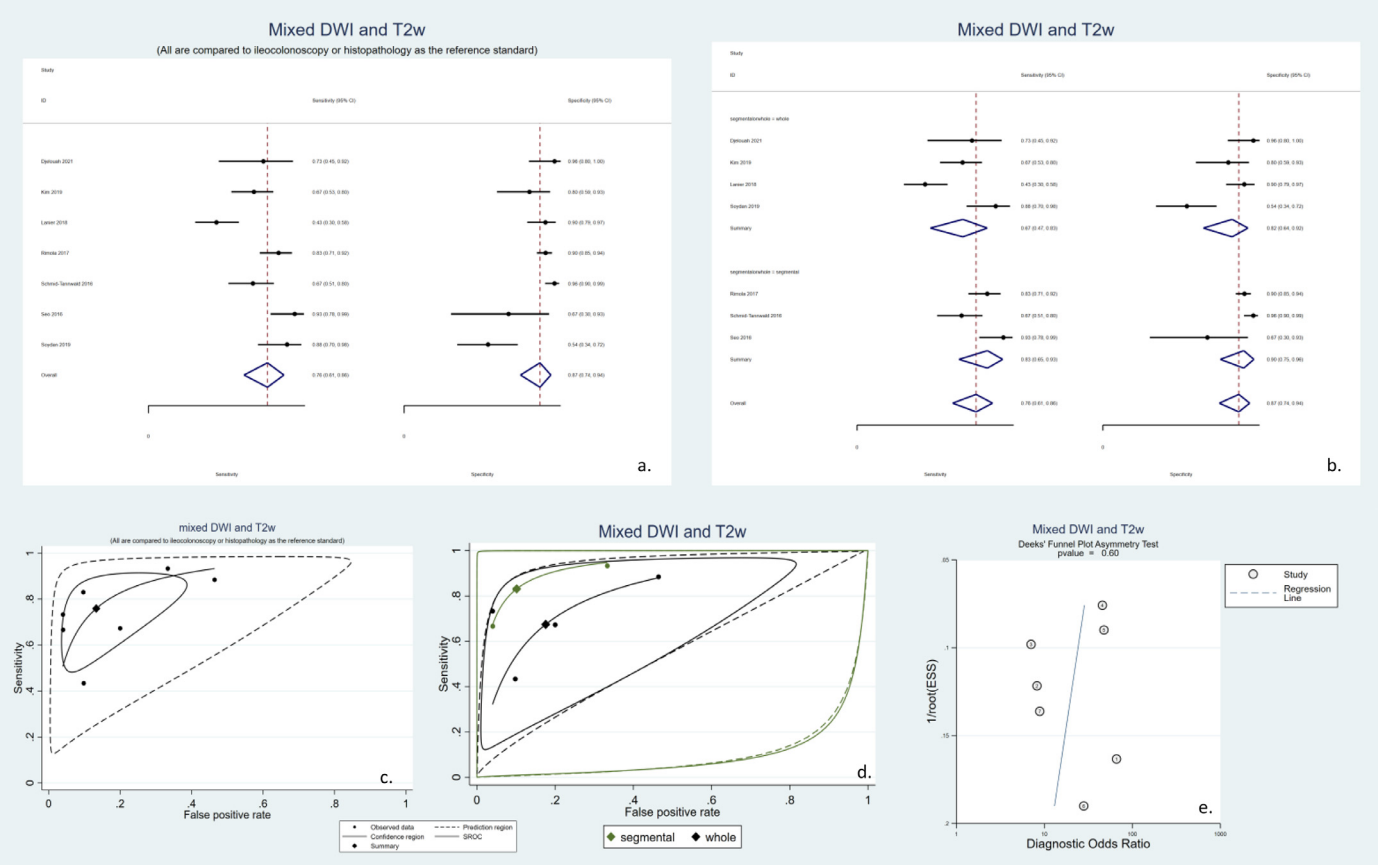
Meta-analysis of all studies of diagnostic test accuracy of all included studies that utilized a mixed protocol consisting of both diffusion-weighted imaging (DWI) and T2-weighted imaging (T2w). a. Forest plot of all mixed protocols with T2w and DWI sequences stratified by the utilized reference test. Due to the fact that all of these studies used histopathology or ileocolonoscopy as the reference standard, there is only one category. b. Forest plot of all mixed protocols with T2w and DWI sequences studies stratified by the study design (segmental or whole-bowel approach) c. Summary receiver operating characteristics (SROC) of all mixed protocols with T2w and DWI sequences stratified by the utilized reference test. Due to the fact that all of these studies used histopathology or ileocolonoscopy as the reference standard, there is only one category. d. SROC of all mixed protocols with T2w and DWI sequences studies stratified by the study design (segmental or whole-bowel approach). e. Deek's funnel plot asymmetry test to investigate publication bias. DWI: diffusion-weighted imaging. SROC: summary receiver operating characteristics. T2w: T2 weighted imaging.

Although different relevant studies have used various methods to combine the two sequences, we have analyzed all of these studies together as a single mixed T2w and DWI protocol. Surprisingly, as demonstrated in Fig. 3, combined T2w and DWI protocols formed an SROC ADC with less diagnostic performance than each DWI and T2w sequence alone in all subgroups except when analyzed based on independent bowel segments. This observation might be caused by different interpretation methods used in T2w+DWI studies and the fact that the number of T2w + DWI studies is relatively low, and the findings in that subgroup might be easily affected by the high heterogeneity. Fig. 8 depicts the results of the meta-analysis results of such combined protocols. The included studies had a pooled sensitivity of 76% (95% CI: 61–86%), a pooled specificity of 87% (95% CI: 74–94%), and high heterogeneity ($I^{2}$ = 93%). It should be noted that all of the included studies that reported diagnostic abilities of combined T2w and DWI sequences used reference tests that were considered gold standards (Fig. 8a, 8c). Interestingly, when grouping the studies based on the segmental or whole-bowel design, it seems that the studies that adopted a segmental approach have a better diagnostic ability, comparable to other alternative tests (Fig. 8b, 8d). As depicted in Fig. 8e, no significant publication bias was observed.

## 4 Discussion

The present study aimed to assess the potential of abbreviated non-contrast MRE protocols in diagnosing active inflammation in Crohn's disease compared to conventional MRE. The conventional protocol for MRE usually consists of T1w CE sequences, which serve as the most crucial sequence in diagnosing active disease, as well as T2w sequences and optional DWI sequences. CE T1w sequences necessitate IV GBCAs, which are undesirable because of contraindications, significant adverse reactions in individuals with renal failure, and the growing evidence of potential Gadolinium accumulation in nerve and bone tissue [15,29]. Furthermore, the current protocols for MRE in CD patients are usually associated with prolonged acquisition times resulting in high cost and low patient compliance. Thus, developing abbreviated protocols with reduced acquisition times while maintaining sufficient diagnostic power is an area of significant interest. Relevant abbreviated MRE protocols include DWI, T2w sequences such as bSSFP/trueFISP, and any other sequence that does not necessitate the use of GBCA, which might also offer a shorter acquisition time. For instance, A previously proposed abbreviated protocol by Cicero et al. has a 35% shorter acquisition time [16]. However, the accuracy and diagnostic capabilities of alternative sequences are still debated, hindering radiologists from developing globally accepted abbreviated protocols for future recommendations.

To develop an abbreviated alternative protocol for CE-MRE, several sequences and modalities can be considered. For instance, T2w sequences, particularly fast T2w sequences, such as bSSFP/trueFISP, can be a suitable choice due to their shorter acquisition time and lower artifact rates. Additionally, CINE motility videos based on fast T2 sequences can also be utilized. Another relevant sequence is DWI, which has been proposed as a non-contrast protocol with high diagnostic accuracy and is already included in some guidelines. Furthermore, innovative MRE sequences such as MTI have the potential to be a subject of future research. To gain a better understanding of the current evidence surrounding an abbreviated MRE protocol, we conducted a systematic search of medical evidence databases. 59 relevant studies were included in the analysis, with DWI and its related techniques, including ADC, being the most frequently investigated sequence. Although the results regarding DWI were highly heterogeneous, they mostly confirmed its diagnostic ability. Other frequently studied sequences included various T2w imaging modalities, which can also be used in combination with DWI to create a comprehensive abbreviated protocol.

We conducted a quantitative synthesis of the studies that provided diagnostic rates by categorizing them into five subgroups based on the sequence used (DWI, ADC, combined T2w and DWI, T2w, and CINE T2w) to provide an objective comparison of each test's performance. Overall, our findings demonstrated significant heterogeneity, which could have been influenced by the reference test and the whole bowel-based or segment-based design of the included studies. Although we attempted to control for these confounding factors, heterogeneity persisted in the majority of groups even after subgroup analyses. This could be due to other factors, including the technical procedure or the location of the examined bowel, which cannot be meta-analyzed due to the limited number of studies available. Despite this, our results showed that DWI and ADC generally had higher diagnostic abilities than their competitors, as illustrated in Fig. 3.

DWI is a technique for calculating the diffusivity of water molecules within the tissue. DWI reflects intestinal wall inflammation, often hyperintense due to restricted water mobility caused by histological alterations, including lymphoid aggregation and increased cellular population [30]. Moreover, ADC mapping measures the magnitude of DWI quantitatively. Several studies have reported promising findings in DWI evaluation, showing a high degree of correlation with findings of the endoscopic evaluation and CE-MRE [31], [32], [33]. We observed high levels of heterogeneity in our meta-analysis, which can be accounted for by differences in reference tests, differences in examination and interpretation methods, and differences in analyzed bowel segments. Chavoshi et al. previously conducted a meta-analysis to assess the accuracy of conventional MRE, reporting significant variability in test sensitivity and specificity across different colon segments [34]. Thus, it is likely that similar differences would exist in the diagnostic performance of DWI modalities as well. According to a recent study, although DWI may be more efficient in detecting lesions in the small bowel, T2 wall thickening could be a more suitable method for identifying colon lesions [35]. our qualitative analysis revealed that DWI is often recommended as an alternative or complementary sequence to the conventional protocol as it exhibits comparable levels of accuracy with the full protocol. In addition, a majority of the studies that were included in our analysis focused on assessing the efficacy of DWI in detecting lesions in the terminal ileum. However, some of the studies did not support using DWI and ADC. For instance, Dillman et al. and Pendsé et al. noted that although significant abnormalities are noticeable in ADC maps, they lack the necessary accuracy for the detection of active disease [36,37]. Similarly, Rimola et al. argued that T2w sequences are adequate for assessing active disease, and the addition of DWI does not improve diagnostic performance [38]. In terms of technical aspects, studies have shown a preference for high b-value imaging over lower b-values [39]. Another study favored sequences that included Spectral pre-saturation with inversion recovery (SPIR) [40].

With regard to potential novel technologies utilizing DWI, recent research has indicated that diffusion Kurtosis imaging (DKI) and intravoxel incoherent motion (IVIM) are alternative techniques that may offer higher levels of accuracy than conventional DWI and hold promise for future use [41,42]. Additionally, Jakob et al. have employed an innovative color-coding program for DWI images, demonstrating favorable results [43]. Moreover, DWI and ADC mapping have been utilized for detecting fibrostenotic complications, which will be discussed in more detail later.

The role of ADC in producing objective assessments in suspected regions of interest (ROIs) has been a subject of interest due to its quantitative nature. Our study found that ADC mapping and quantitative analysis generally provided excellent accuracy, even better than DWI alone, likely due to its objective design. However, there is significant disagreement between studies regarding the optimal threshold value for ADC. The optimal ADC threshold for the maximum AUC ranged from 0.86 in one study [42] to as high as 2.1 in another study [44]. Nevertheless, the majority of studies included in our analysis proposed a threshold value of approximately 1.4, as summarized in Table 2. While ADC has shown promise in diagnosing bowel wall fibrosis [45,46], its diagnostic accuracy outperformed by MTI, another MR modality specifically designed for assessing bowel wall fibrosis, as we will discuss later in this article [22].

T2w sequences have gained interest due to their fast acquisition times and reduced susceptibility to various forms of artifacts, such as those caused by intestinal gas and motility. The HASTE sequence, which is a commonly used T2w sequence in MRE protocols, is gradually being replaced by newer sequences such as TSE and bSSFP. These more recently introduced sequences, including trueFISP, FIESTA, or balanced fast field-echo (bFFE), are available in various devices and can be acquired without the use of antiperistaltic drugs, making them more convenient and practical [18]. In addition, the short acquisition time of these sequences enables the assessment of bowel wall motility using CINE sequences, which can be further processed using specialized software to calculate bowel wall motility indices. The primary signs of active inflammation in T2w sequences are wall thickening, T2 hyperintensity, and vascular engagement, referred to as comb sign [18]. Accurate interpretation of these signs can facilitate the diagnosis of bowel wall inflammation and even differentiate the three phenotypes of CD without the need for potentially harmful GBCAs [47].

Among the included studies, T2w sequences have been extensively studied, either independently or as part of abbreviated protocols combined with DWI, and despite some discrepancies, most studies have recommended their use. However, based on our quantitative synthesis, T2w sequences showed lower diagnostic abilities compared to DWI. Interestingly, the AUC under the SROC curve of mixed T2w and DWI sequences was found to be smaller than that of DWI alone, which was a surprising result. Nonetheless, these findings may have been influenced by factors such as the type of analysis employed. Additionally, as mentioned earlier, repeated acquisition of fast T2w images such as bSSFP can generate CINE motility data, which can be visually or quantitatively analyzed. Typically, a decrease in motility is associated with bowel wall pathologies, such as fibrosis or inflammation [48,49]. However, our meta-analysis indicated that this technique's accuracy might not be sufficient for primary assessment. Nonetheless, as it is safe and has a short acquisition time, it can be included in abbreviated protocols as a supplementary modality.

Although the primary focus of MRE in CD is on detecting inflammatory lesions, identifying complications is also crucial. CE-MRE has been shown to have good accuracy in identifying such lesions [50], and recent studies have analyzed the diagnostic capabilities of abbreviated protocols in this regard. While some studies show the strong consensus of T2w-based bSSFP/true FISP with CE-MRE in identifying all three main complications [51], others demonstrate the lower ability of abbreviated protocols in identifying penetrating disease, indicating the need for CE-MRE [52]. Fibrostenotic lesions are the most common complication of CD and can be challenging to diagnose accurately. Abbreviated T2w and DWI protocols depict restricted diffusion and increased wall thickness in fibrostenotic disease, respectively [45]. Therefore, they cannot diagnose fibrosis in a bowel affected by multiple inflammatory lesions. To address this issue, MTI has emerged as a promising imaging technique for diagnosing bowel fibrosis with excellent accuracy and specificity [53,54]. Moreover, MTI-based magnetization transfer ratio (MTR) measurements offer a quantitative approach to distinguishing between fibrosis and inflammation [22]. Recent research has proposed that standardized MTR may have superior diagnostic power for detecting fibrotic lesions compared to normalized and simple MTR [55].

### 4.1 Limitations

Our study has certain limitations inherent to the work's type and methodology. Firstly, there was a significant amount of heterogeneity in the included studies, making us unable to interpret most of the existing evidence with quantitative and objective measures. The existing relevant studies can be divided into many subgroups with limited studies. Furthermore, each study has applied different protocols and sequences specific to the protocols in their center, making it more difficult to compare the results. The reference standards and the analysis approaches are also potential confounding factors.

Moreover, inherent to most studies evaluating the diagnostic abilities of radiologic tests is the fact that it is highly dependent on the expertise and experiences of the radiologist. For instance, one of our included studies demonstrates a high difference between the accuracy of the tests when examined by a senior radiologist compared to a junior one, making their proposed abbreviated protocol only accurate enough for senior radiologists [56]. Therefore, it is a legitimate concern in research of a similar nature that administering tests in referral centers and by highly qualified professionals may induce bias by overestimating the accuracy.

## 5 Conclusion

In this study, we have critically reviewed more than 50 studies concerning the feasibility of abbreviated protocols for MRE. Despite the considerable heterogeneity observed among the reviewed studies, our findings suggest that unenhanced MRE can serve as a promising alternative to traditional contrast-enhanced MRE sequences for detecting active inflammation in patients with CD. In particular, we found that DWI and ADC-based sequences exhibit the highest accuracy among alternative sequences, making them suitable candidates for inclusion in abbreviated protocols. Additionally, low acquisition time T2w sequences can be utilized alongside DWI-based images to further enhance accuracy.

However, it is worth noting that DWI and T2w sequences may have limited diagnostic power for CD complications, particularly penetrating complications. However, our review suggests that MTI has a high discrimination power for detecting fibrostenotic disease. MTI can be acquired and analyzed alongside ADC maps to differentiate between fibrosis and active inflammation in patients with a high clinical suspicion of fibrosis.

According to the reviewed evidence and discussed matters, we propose using an abbreviated MRE protocol based on DWI, ADC, and low acquisition time T2w imaging sequences, including trueFISP for routine screening of CD patients for active inflammation and disease relapse. In case of abnormal findings in the abbreviated protocol, CE-MRE should be indicated in the second step for confirmation and searching for CD complications. CE-MRE should also be indicated in case of clinical suspicion for penetrating disease. Due to its excellent diagnostic power in detecting fibrosis, MTI should be indicated in case of clinical suspicion for fibrostenotic disease. Adopting such a protocol will potentially decrease the costs, increase patient compliance, and minimize the administration of GBCAs, protecting the patients from short and long-term side effects of repeated CBCA exposure.

## Financial aid

We declare that this paper has not received any financial aid or grant from any government or non-government organization**.**

## CRediT authorship contribution statement

**Payam Jannatdoust:** Conceptualization, Data curation, Formal analysis, Investigation, Methodology, Software, Visualization, Writing – original draft, Writing – review & editing. **Parya Valizadeh:** Data curation, Formal analysis, Writing – original draft, Writing – review & editing. **Mahshad Razaghi:** Data curation, Writing – original draft, Writing – review & editing. **Maedeh Rouzbahani:** Data curation, Writing – review & editing. **Amirbahador Abbasi:** Data curation, Writing – review & editing. **Arvin Arian:** Conceptualization, Methodology, Project administration, Resources, Supervision, Validation.

## Declaration of Competing Interest

The authors declare that they have no known competing financial interests or personal relationships that could have appeared to influence the work reported in this paper.
